# Addressing Community Health Needs through the Development of a Student-Led Community Health Fair

**DOI:** 10.15694/mep.2020.000175.1

**Published:** 2020-08-26

**Authors:** Jacob Ritchie, Lauren Tyler, T.J. Wenzel, Alyssa A. Guo, Lauren Fowler

**Affiliations:** 1 University of South Carolina School of Medicine Greenville

**Keywords:** Community outreach, active citizenship, health disparities, medical student leadership

## Abstract

This article was migrated. The article was marked as recommended.

Problem:

Based on a specific community benefit analysis of Greenville, South Carolina, we identified the Dunean community with its increased prevalence of health inequities with respect to access to health care, poverty burden, and disease mortality on a county, state, and national level. The Dunean community’s data reflect poorer health outcomes in terms of disease and unhealthy lifestyle as well as inadequate access to medical resources compared to other communities in South Carolina.

Approach:

Students, residents, attendings, faculty, and staff from the University of South Carolina School of Medicine Greenville (UofSC SOMG) formed a task force to engage the community and combat the root causes of diseases. This task force built partnerships with community leaders to create *Root Cause*, a monthly health event designed to bring community members to a unified space, share a free community dinner, and provide a wide range of health and wellness resources to educate and inspire them to make healthy lifestyle choices.

**Outcomes**:

This report describes the formation of the community engagement task force and execution of *Root Cause.* In five *Root Cause* events, we partnered with 36 community agencies and our academic health center partners who shared their resources, served 207 Dunean neighborhood members, and facilitated 1,237 total interactions between community members and partners.

Conclusion:

Under the *Root Cause* model, medical students and neighborhood partners have initiated a trusted, bidirectional dialogue to determine their specific needs with the desire to positively transform the health and wellness of the Dunean community. Our data suggests that based on our efforts, the neighborhood of Dunean, SC increased community cohesiveness and improved perceptions of access to health care. Additionally, participating medical students advanced their understanding of social health and economic challenges which helped to facilitate their development along the active citizen continuum, as well as increase empathy for their future patients in the local community.

## Problem

Since the inaugural class of the University of South Carolina School of Medicine Greenville (UofSC SOMG) matriculated in the Fall of 2012, the students, faculty, and staff have slowly introduced community engagement into the framework and weekly proceedings of the medical school. This allowed for countless partnerships and volunteering opportunities that benefited the communities surrounding the medical school and Prisma Health’s Greenville Memorial Medical Campus. However, we discovered a greater need for involvement in our communities that had not yet been addressed and for a transition from volunteerism, which lacks social context, to culturally competent active citizenship.

**Figure 1.**
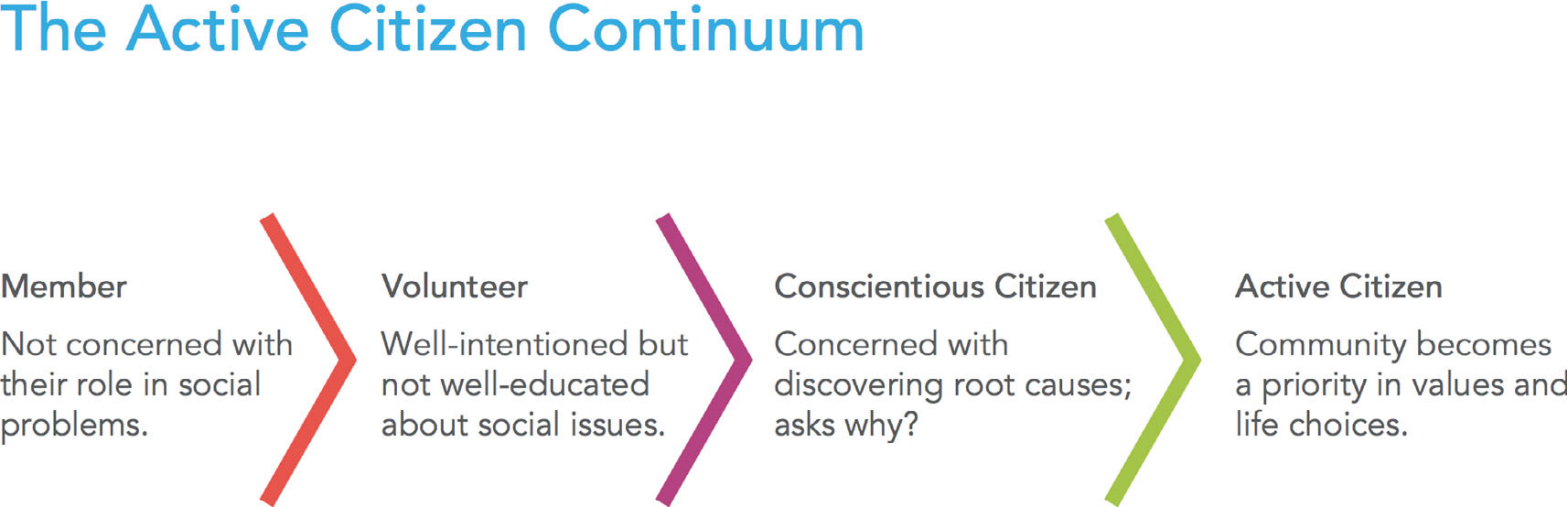
The Framework for the Active Citizen Continuum (figure used with permission from “Break Away: The Alternative Breaks Connection, Inc”).

On a statewide level, a 2017 Annual Report from American Health Rankings showed that South Carolina ranked 44 out of 51 states for population health, with high premature death and high rates of diabetes leading the list of concerns (DHEC.SC.gov, 2017). Disparity in health status rank was the 14th highest in the United States ( United Health Foundation, 2017). According to a 2016 Greenville Health System (now Prisma Health) Community Health Needs Assessment, eight main priority issues were identified that applied to Greenville/Pickens, Oconee, and Laurens counties, counties in which the hospital system provides medical care: unintentional injury, heart disease mortality, obesity, alcohol & drug abuse, suicide mortality, diabetes, cancer mortality and mental health ( GHS.org, 2016). In regard to Greenville county, Furman University’s 2018 Needs Assessment found 10 neighborhoods that were identified based on highest family poverty levels, high percentage of minors per capita, and high percentage of single female-headed households (Furman.edu, 2018). The data from these assessments allowed us to find specific geographic communities at most risk for suboptimal medical outcomes.

When taking each analysis into account and accessibility for UofSC SOMG and its budding active citizens, the data demonstrated that the community adjacent to UofSC SOMG, Dunean, was an at-risk community with high rates of poverty, diabetes, and obesity with low rates of perceived access to health care. In 2017, Dunean had a population of 3,710 people (58.4% Caucasian, 34.4% African American, 2.6% Hispanic or Latino) ( DataUSA, 2017). The median household income was $28,866, with 23% of residents living in poverty ( DataUSA, 2017). Many of these statewide problems are exacerbated in the Upstate of South Carolina, and the Dunean community performs lower than much of the region on access to health care and health measures ( GHS.org, 2016). The needs of the Dunean community inspired us to work within the missions of both UofSC SOMG and Prisma Health to develop a program focused on addressing the community’s needs and to improve the health of the communities we serve. Instead of traveling across Greenville County for our inceptive event, we wanted to address the health disparities within our own backyard first.

## Approach

In the summer of 2018, the Associate Dean of Academic Affairs, Dr. Angela Sharkey, formed a task force composed of Greenville Health System (now Prisma Health) clinically practicing physicians and UofSC SOMG Community Outreach student leaders, faculty, and staff. The purpose of this task force was to explore how to shift student, faculty, and staff volunteerism towards a focus on active citizenship and to impact meaningful change in the health of our community. To accomplish this goal, we recognized the need to build trusting relationships with our community and to develop mechanisms for bidirectional decision-making. After a year of discussion and planning, the task force decided to proceed with a monthly health and public services fair, *Root Cause*, that would address many of the health disparities that were identified in the numerous needs assessments. Our goal was to ensure that we were making a noticeable and reproducible change within the community in which we chose to start our initiative, while also eventually leaving the community better than when we arrived. In forming this task force, we also sought to reflect the UofSC SOMG Community Outreach group mission statement: “To positively impact and address a diverse array of health disparities that are present within the Greenville community, the Upstate, and South Carolina while maintaining an intentional, collaborative, and engaging experience for our members.”

Along with taking the specific needs into account of Greenville County as a whole, the UofSC SOMG Community Engagement Task Force met with members of the Dunean community (the Neighborhood Association, the Historical Society, United Way, the sheriff’s department, and members of two church congregations) to identify specific needs within the community that could be addressed by *Root Cause.* Two main concerns of the community were lack of community cohesiveness and community members’ barriers to health care and preventative care access. *Root Cause* was developed to address these community specific needs in addition to incorporating the needs that were identified by the various needs assessments.

Each month, *Root Cause* engages UofSC SOMG students and faculty, Prisma Health physicians, residents, and staff, and community partners with our neighbors in Dunean and to address the root causes of disease by increasing access to health care and promoting healthy lifestyle choices. The *Root Cause* initiative is an interdisciplinary collaboration of more than 36 community partners that encompasses 5 main categories: Healthy Meals & Nutrition, Student Interest Groups, Preventative Initiatives, Medical Clinics, and Advocacy/Informative resources. Topics and community partners rotate on a monthly basis to ensure adequate and appropriate representation in each respective category. We also seek to connect medical students and residents with community members to better understand their needs, reduce medical student and resident burnout early in their medical education through community engagement experiences, give back to our local community, and provide a platform for greater community cohesiveness. Our goal is that through *Root Cause*, we are “growing a healthier Greenville” through:

• COMMUNITY: Positively influencing a stronger, collaborative and connected community
• ACCESS: Improving access to valuable health care resources
• RESEARCH: Research to build sustainable change
• EDUCATION: Building new knowledge for our participants

### Community

We seek to create a safe and collaborative space for the community to come together, share a free healthy meal, and learn about health and wellness resources available to them. Since transportation may be a factor in access for community members, we determined the best location for our monthly health fair would be in the epicenter of the Dunean community, at either the Leaf Institute (formerly Dunean United Methodist Church) or the BI-LO grocery store plaza parking lot, which is in close proximity to more than 3 bus stops and 2 main roadways. These locations provide access to a wider community pool that could benefit from our events. During this event, we also measure participants’ perceptions of their community connection to investigate this change over time.

### Access

Each month, we address the health disparities identified by the Greenville County benefit analyses along with the direct requests from the Dunean community. To address the decreased perception of access to health care, we host a number of related community partners that provide resources for the community members. We encourage participants to sign up for doctor’s appointments through Prisma Health’s internal medicine, pediatrics, OB/GYN and family medicine clinics, giving access to free insurance through AccessHealth and SC Thrive, and allowing community members to visit the Prisma Health Mobile Clinic. We also provided resources to have blood pressure and blood glucose levels checked by nurses from our internal medicine clinics and recommend that community members learn more about diabetes screenings via the Prisma Health and Clemson diabetes educators. We address the health disparities of obesity, heart disease mortality, and diabetes by providing a free healthy meal from Project Host, healthy cooking demonstrations by a chef involved with UofSC SOMG and the Greenville Technical College Culinary Institute, sign-ups for monthly produce boxes from FoodShare Greenville, and various student interest groups from Furman and UofSC SOMG that discuss healthy alternatives in the form of food and exercise. We attend to the disparity of unintentional injury by involving community partners that raise awareness about gun safety such as Be SMART and drug & alcohol prevention provided by FAVOR Greenville, the Phoenix Center, and Prisma Health Addiction and Recovery. In order to address suicide mortality and mental health, we have involved the Emergency Medicine and Psychiatry physicians and residents from Prisma Health along with the corresponding student interest groups from UofSC SOMG. With targeted foci each month, the community partners are able to assess the needs of the community by gauging interest. We will be looking at the impact these resources make on Prisma Health emergency department visits and the appointments that are made at *Root Cause* and ultimately attended.

### Research

The Community Engagement Task Force identified the lack of research articles that discuss the impact of community engagement on medical students and how the community is impacted by students at a medical school. We have multiple IRB-approved research projects in place that are currently collecting data to answer questions such as, “Does engaging with the UofSC SOMG and community partners through a public health fair increase awareness of access to health care and availability of medical services within and surrounding their community?”, “Does this increased awareness result in increased clinician appointments and decreased emergency room visits?”, and “Does engaging with the community surrounding the medical school increase diversity and inclusivity and decrease student burnout as the students interact with their future patient population?”.

### Education

The entire health fair format allows for the transfer of new information, recipes, resources, common health issues, and much more. The UofSC SOMG curriculum emphasizes health maintenance and preventative care in an effort to reduce individuals’ need for episodic care. This curricular commitment showcases one part of our school’s deeply embedded culture, Lifestyle Medicine, and our goal is to share this knowledge with our Dunean neighbors. We are assessing the knowledge participants gained as a result of participating in *Root Cause.* Each one of our educational materials focuses on how access to health care can affect the whole family. We are working to help educate the community about opportunities for healthy lifestyles and access to health care, but *Root Cause* also serves as an educational opportunity for the students at the medical school to get to know their future patients. This is a transformational time for a medical student, and this model is an opportunity for them to leave the structured learning environment of the classroom to learn about current issues that impact their own community. They are learning about the social determinants of health and how they impact the health of a community.

Medical students stereotypically learn by “see one, do one, teach one.” *Root Cause* seeks for the students at UofSC SOMG to **see** people where they are, rather than only in an office. The future physicians who have a better understanding of the health inequities plaguing their community will ultimately be better doctors because of it. We strive to have our students listen to their community’s needs and respond as active citizens. *Root Cause* seeks for the students at UofSC SOMG to **do** good and be an advocate for initiatives that improve patient health and health of the community. *Root Cause* seeks for the students at UofSC SOMG to **teach** their fellow neighbors how to live their healthiest lives by being a champion for high-value, evidence-based care, as well as culturally appropriate and community-specific care.

## Future Direction

The early data gathered by our group is encouraging, and we look forward to building on what we have learned to enhance the benefit of each *Root Cause* fair for its participants and attendees. In the immediate future, one of our large areas of focus will be on increased advertising and subsequent attendance at each fair. These additional advertising efforts will be tailored to focus on the Dunean community, in order to reach and increase participation from our target population. Such advertising initiatives include marketing on radio stations, local church bulletins, and strategically placing yard signs in heavily traveled areas to name a few. A larger response from the community members will enhance the effect of the initiative, as well as the validity of the data we collect. The number of held fairs for the 2019-2020 academic year was decreased due to weather and the COVID-19 crisis, but we plan to resume the fairs as soon as allowed and have secured their location for the upcoming year. We plan to continue to focus our advertising efforts on reaching out to schools and organizations in the Dunean community. We also plan to continue to solicit the participation of each incoming class of medical students, disseminating information via school-wide email and newsletters, as well as presenting it in initial Orientation sessions. Our participation numbers have been substantial, and this will hopefully continue as the awareness of our initiative grows.

It will be difficult to glean the full impact of *Root Cause* on both the participating medical students and community members until more time has passed and more data has been collected. For the students, initial results have indicated that participation has made them more socially aware and engaged citizens, but its effects on burnout and inclusivity in their eventual practice of medicine are yet to be seen. We are currently assessing the short-term impact of participating in *Root Cause* on the students’ ideas of equity, diversity, and inclusivity. This will continue to be analyzed throughout their participation. There are also possibilities for examining more longitudinal effects on their medical careers, such as examining the role *Root Cause* played in their future choice of specialty and geographical region of practice.

The initial data also supports an increase in community cohesiveness due to *Root Cause*, based on the responses of participating community members and volunteers. We will look to expand on this data, getting a better idea of what this means from the side of the participating community organizations. This will require developing separate surveys for the community organizations that have participated in each event, in order to identify any changes in their perspective. While we have documented the number of their interactions, we are interested in examining how their participation has impacted their awareness of other organizations in the community and their familiarity with the community’s members and their unique needs. This could be applicable to both participating health care and non-health care organizations.

From a preventative medicine approach, *Root Cause* has been working on introducing healthy lifestyle medicine education while taking into consideration the financial barriers to healthy eating. In South Carolina, 11.7% of households remain food insecure ( United Health Foundation, 2019). Food insecurity is defined as limited or uncertain availability of nutritionally adequate foods or uncertain ability to acquire food in a socially acceptable manner ( Bickel *et al.*, 2000). This is one of the consequences of low socioeconomic status and may reflect dietary habits involving the type of products that are purchased and the methods they are prepared, and ultimately, identifying and combating food insecurity may improve health outcomes in patients with chronic diseases, such as diabetes ( Mendoza *et al.*, 2018). Due to the current COVID-19 pandemic, as more research becomes published, the food insecurity problem may be further compounded by unhealthy dietary changes resulting from elevated stress and distress due to limited access and financial restraints ( Naja and Hamadeh, 2020). To tackle food insecurity in our community, *Root Cause* has been conducting grocery tours that are focused on promoting healthier diets, dispelling myths that healthy eating is expensive and unattainable on a budget. While there are grocery store tours available in the Greenville, South Carolina area, most of their target audiences are patients with chronic diseases. At *Root Cause*, we are seeking to create a sustainable program for the entire community at large, including the underserved, and allow interested community members to learn about healthy eating options while staying on a budget. With the ongoing COVID-19 crisis, the need is greater than ever. As soon as we are able to return to the community, we will continue to hold personalized grocery store tours tailored to an individual’s needs and preferences, and these tours have the option to be in person or be showcased using a 360 video with virtual reality goggles. Our new campaign, and the associated research project to track the tours’ effectiveness, aims to be a multi-pronged approach seeking to positively influence grocery shopping and dietary habits in community members, especially those who are experiencing food insecurity.

We will also continue to monitor the effects of *Root Cause* participation on Prisma Health emergency department visits, along with the appointments made (and subsequently attended) by community members at each fair. Increased primary care physician appointments and decreased non-emergency visits to the Emergency Department would indicate that members of the community are aware of and are making use of health care resources available to them. We hope that there will be direct evidence of a closing of the identified gaps in the health care education and needs of the Dunean community.

The ultimate goal of the *Root Cause* task force and leadership team is to develop a set of best practices and translate what was effective in this initial effort to other communities with similar needs. However, our immediate objective is ensuring that we have addressed the needs of the Dunean community. The task force never intended for this to be a series of quick, blanket ventures into various communities, but instead, we hope to create sustainable, positive changes in addressing the unique needs of our community of focus. In order to uphold this vision, the task force must take the time, especially in this initial community, to learn from the data. We must verify the Dunean needs, identified from the outset of the project, are being met. We plan to continuously reassess those needs through our bidirectional relationship with both the Dunean community and the community partners we have established through *Root Cause.* This includes contacting community partners regularly to determine any additional needs they have identified through their participation or any gaps they noticed while addressing our previously identified needs. Only when we are satisfied with the progress toward addressing Dunean’s needs will we begin to look for the next community in which to establish *Root Cause.* At that time, we will gather an updated Needs Assessment list. Ideally, any communities appearing on both the updated and original lists would be considered priority, as their needs are apparent as a persistent issue. Once the new community of focus is identified and agreed upon by the task force, we will once again work with various community members and organizations to identify what they perceive to be their greatest needs. We can then incorporate these into the results of the Needs Assessment, to best tailor the organization of their *Root Cause* fairs. We are confident that our experience and data will only serve to improve the effectiveness of the events as we move forward.

## Conclusions and Recommendations

**Figure 2.**
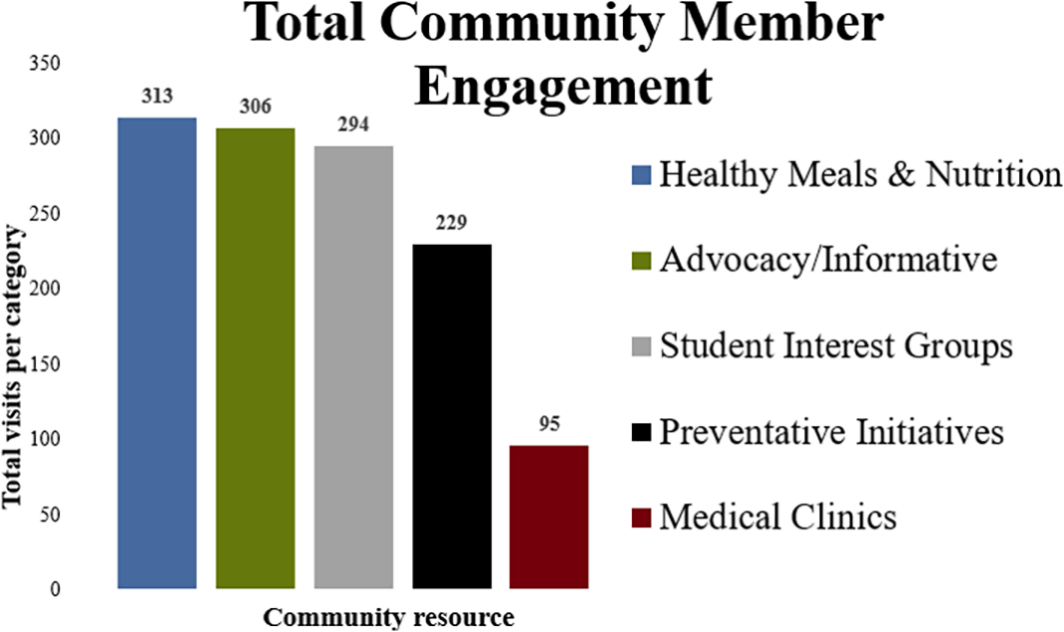
Number of Community Members Accessing Community Partner Resources Based on Category.

Outcomes:

Based on the five *Root Cause* events that have been hosted, approximately 207 community members have attended, with 74 participants completing the surveys. Survey results indicate that those community members who have attended more *Root Cause* events rate their community cohesiveness as higher, r(68) =.220 (p<0.05). Community partners have steadily increased at each event, with 36 community partners now having engaged members of the Dunean community through *Root Cause.* As part of their outreach, community partners have had 1,237 total interactions with Dunean community members. The numbers of community members accessing community partner resources can be found in Table 1 and Figure 2.

**Table 1.** Community Member Participations and Educational Opportunities for *Root Cause.*

**Number of Community Members**	**Skill/Educational Materials**
122	Learned hands-only CPR
514	Educated about healthy lifestyle, exercise, and cost-effective meals
99	Learned about gun safety
132	Received information on addiction and recovery
75	Learned about or left a Root Cause event with an appointment with a Primary Care Physician

Community partner tables that received the highest attendance by community members are those related to food (healthy cooking demonstrations, free meals, etc.), overall advocacy & informative booths, and interactive activities from student interest groups (CPR training, virtual reality grocery tour, blood pressure checks, etc.).

**Figure 3.**
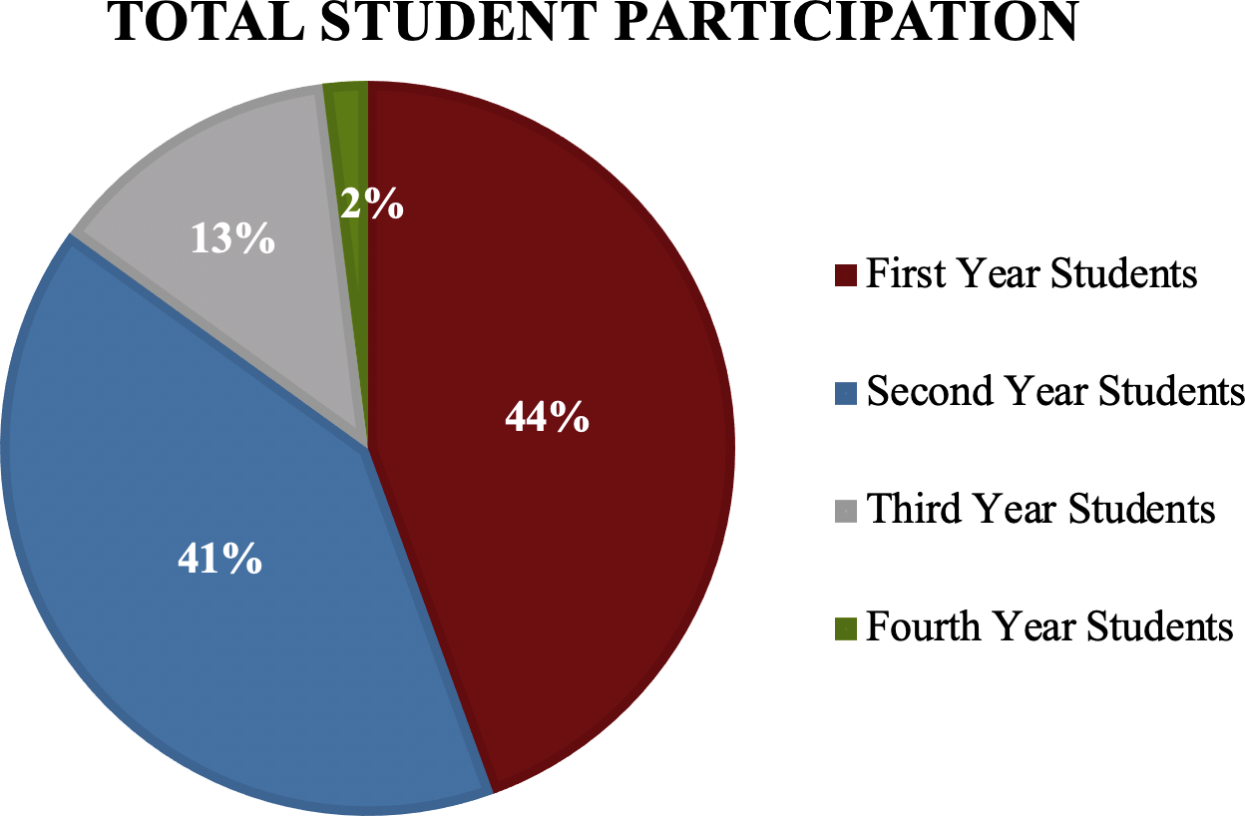
Percentage of Student Volunteers Based on Year in Medical School.

153 medical students participated in *Root Cause* through volunteering with 85% of students being first and second year medical students ( Figure 3). Students were able to volunteer in the following capacities: education through a student interest group, marketing, photography, distributing surveys to attendees, and welcoming attendees to the events. 44 student surveys were completed over the five *Root Cause* events, and results from the student surveys showed that more first year medical students completed the survey (47.7%) than any other year. In addition, results demonstrated that students at UofSC SOMG advanced on the active citizenship scale from volunteer to conscious citizen after getting involved in the community and progressing through the school year.

Results from the surveys indicate that the collaboration among the medical school, community partners, and community members to develop a monthly health fair increased community cohesiveness and perceptions of access to health care. Early data analysis showed that participation in *Root Cause* advanced our students forward on the active citizenship continuum as shown in Figure 1. In addition, the more medical students participated in *Root Cause* events, the more their participation increased their empathy for their future patients in the community. Preliminary results indicate that more research and student participation will need to be done to continue to see significant results in burnout and medical student perceptions of equity, diversity, and inclusivity. In addition, this collaboration provided a forum for community partners to make connections in the Dunean community, a community that they may not have had access to otherwise. This *Root Cause* health fair model can thus be used to promote access to health and healthy behaviors in communities and to increase community impact by partnering community partners with a medical school.

Under the health fair model, *Root Cause* was beneficial for students, faculty, and physicians to work collaboratively in order to ensure all aspects of the medical team were represented. The faculty and physicians gave the task force legitimacy, mentorship, and contacts to specific resources that would have otherwise been difficult to obtain for the students. The students had the opportunity to learn and work side-by-side with their teachers and attendings in starting an initiative that benefited the surrounding community. The *Root Cause* model also gives the medical students leadership skills in interacting with their local community leaders and members.

The main factors determining the replicability of this model must reflect community-specific needs and cultural competencies. There must be a bidirectional dialogue with the community leaders from the inception of planning in order to appropriately impact the neighborhood. While many partners have shown interest in being involved with this monthly health event model, priority must be given for the resources that are needed and requested by the community. The time of day, day of the week, and location of the event are all variables that were discussed and determined by our prioritized, core partners. Thankfully, we have been able to accommodate the majority of community partners into our events that have shown interest to date due to the flexibility of the task force and partners.

Lastly, a vital piece of our initiative is the student volunteers giving their time each month and planning in the interim. Medical students have an innate motivational drive to complete difficult tasks even when others stop. This attribute makes them successful in many aspects of life, especially in medical school. The UofSC SOMG community engagement task force has been able to access that motivational drive and harness the students’ energy to learn about the health disparities and needs of the community. This allows the students to get to know their future patient population on a deeper level, rather than reading it from a textbook. Students are impacting the lives of their neighbors and preventing negative health outcomes before they graduate medical school. Ultimately, *Root Cause* is helping to cultivate the transition from uninformed student volunteerism to culturally competent active citizens within our own local communities.

## Take Home Messages

• Community health fairs organized by medical schools, such as *Root Cause*, can provide a culturally competent platform for community partners to address health disparities in local neighborhoods.
• Medical students can serve as leaders in developing bidirectional engagement relationships with members of their community.
• Medical students can enhance their empathy, resiliency, and multicultural attitudes through community engagement, thus providing better patient care and improved patient satisfaction.
• Medical schools, their students, and their faculty/staff can impact their communities to increase community cohesiveness, promote healthy lifestyle choices, and improve perceptions of access to health care.

## Notes On Contributors

**Jacob Ritchie** is a fourth-year medical student at the University of South Carolina School of Medicine Greenville, served as the 2018-2019 Community Outreach Student Director, and is a founding member of the Community Engagement Task Force and the Root Cause initiative.

**Lauren Tyler** is a third-year medical student at the University of South Carolina School of Medicine Greenville who served as the 2019-2020 Community Outreach Student Director and the 2019-2020 Root Cause Director.

**T.J. Wenzel** is a second-year medical student at the University of South Carolina School of Medicine Greenville and is currently serving as the 2020-2021 Community Outreach Student Director.

**Alyssa Guo** is a second-year medical student at the University of South Carolina School of Medicine Greenville, served as the 2019-2020 Root Cause Assistant Director, and is currently serving as the 2020-2021 Root Cause Director.

**Lauren A. Fowler, PhD**, is an Associate Professor of Neuroscience in the Department of Biomedical Sciences at the University of South Carolina School of Medicine Greenville and a member of the Community Engagement Task Force. ORCID iD: https://orcid.org/0000-0002-3494-6334
