# Supplementary material for: Addressing Community Health Needs through the Development of a Student-Led Community Health Fair
Source: MedEdPublish (2016). 2020 Aug 26;9:175. [Version 1] doi: 10.15694/mep.2020.000175.1 (PMC10702671; doi:10.15694/mep.2020.000175.1)
Supplement: Peer review history

# Reviewer report: Reviewer response for version 1

**DOI:** 10.21956/mep.20087.r30932

**Published:** 2021-02-11

**Authors:** Richard Hays

**Affiliations:** 1 James Cook University

This review has been migrated. The reviewer awarded 4 stars out of 5 This article describes a very useful initiative that has benefits for both students and the wider community. Students benefit from listening to the communities they may serve and understanding their health care needs, while the community benefits from understanding better the health care system and how they can get the most from it. The article is well written and the messages are clear. I recommend that students and faculty read this and see if the model is adaptable to their context. There are other community-engagement projects in medical schools - 'mini-med school', 'teddy bear hospital' are two that are reasonably common. If a second version is contemplated, I would suggest that the conclusion and recommendations be separated. A conclusion should really be just a paragraph that provides a clear and concise statement about the findings and the implications. I look forward to seeing further contributions on the imp1act of this initiative on both the community and participating students.

Reviewer Expertise:

NA

No decision status is available

# Reviewer report: Reviewer response for version 1

**DOI:** 10.21956/mep.20087.r30933

**Published:** 2020-10-01

**Authors:** Ken Masters

**Affiliations:** 1 Sultan Qaboos University

This review has been migrated. The reviewer awarded 5 stars out of 5 This paper describes a project that seeks to address community health needs through the development of a student-led community health fair.The paper is a really wonderful read, and should be an inspiration to all involved in healthcare education, particularly those located in under-served communities. Although it focuses on the single project in a single area with very specific characteristics, it does make the point of showing the essential principles, beginning with understanding the community’s needs and then working from there into a very broad range of activities. The significant drop-off of student engagement across the students’ years is troubling, but, unfortunately, expected. One hopes that this is mostly because of workload pressure; unfortunately, the number of international studies that indicate the drop in empathy across medical students’ years of study would imply that that is at least an influencing factor. Given that the results of this project indicate a positive impact on student empathy, one may hope that, while the disparity will always exist, it will not always be as extreme. A possible solution may be to seek tasks that require 4th year student expertise, but do not demand so much time. Alternately, if the later curriculum has self-study/research modules, then specifically-tailored modules can be created.The impact of COVID on the project is also unfortunate – and sadly (and troublingly ironically), at a time and in a place where a project like this is most needed.I look forward to reading further research articles on this project.

Reviewer Expertise:

NA

No decision status is available
